# A case report of multi-step management of extracranial carotid artery aneurysm and carotid-cavernous fistula combination in patients

**DOI:** 10.3389/fneur.2023.1120786

**Published:** 2023-04-06

**Authors:** Chingiz Nurimanov, Yerbol Makhambetov, Assylbek Kaliyev, Serik Akshulakov

**Affiliations:** Department of Vascular and Functional Neurosurgery, National Center for Neurosurgery, Astana, Kazakhstan

**Keywords:** case report, fistula, aneurysm, endovascular embolization, stenting

## Abstract

Extracranial carotid artery aneurysms (ECAA) and carotid-cavernous fistulas (CCF) are rare arterial pathologies with severe complications and increased risk of mortality. The optimal treatment approach for this combined condition is a topic of debate among neurosurgeons and neuroradiologists, and a standardized treatment protocol has yet to be established. The aim of this case report was to demonstrate the management of a rare combination of ECAA and CCF in patients. The treatment strategy included a two-step procedure of endovascular embolization of CCF followed by dual antiplatelet therapy and endovascular stenting of an aneurysm. Control angiograms showed the exclusion of an aneurysm from the blood circulation and CCF symptoms were resolved.

## 1. Introduction

Extracranial carotid artery aneurysm (thereafter – ECAA) is a rare vascular disease that constitutes 1.5–4% of all cerebral aneurysms (1, 2). Early diagnosis and treatment of ECAA are important as many cases remain asymptomatic until thromboembolic events, cranial nerve compression, or a rupture of an aneurysm. Treatments for this condition represent 0.2–5% of carotid procedures according to the institutional reports (3). Carotid cavernous fistulas (thereafter – CCF) are abnormal communication of the cavernous sinus and the previously normal carotid arterial system (4). The combination of CCF and ECAA is an extremely rare condition that often requires neurosurgical intervention. In this report, we present two clinical cases of this rare combination of pathologies in patients, highlighting the challenges involved in diagnosing and treating this complex condition.

## 2. Case reports

### 2.1. Case 1

**Figure 1:**
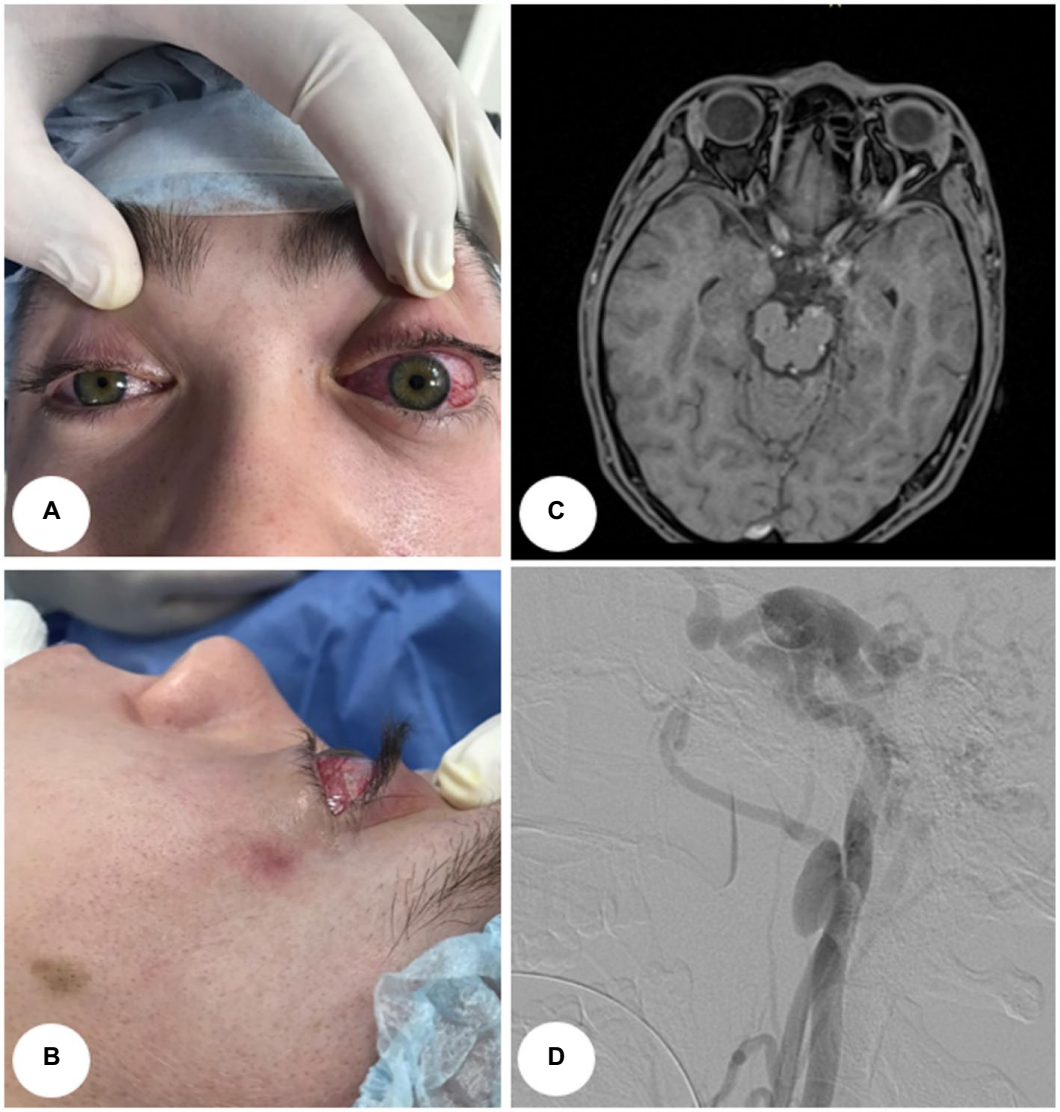
A visual manifestation and instrumental examinations of Case 1. **(A)** Anterior view. **(B)** Lateral view. **(C)** Axial MRI shows dilatation of the superior ophthalmic vein in a patient with left side carotid-cavernous fistula. **(D)** Digital subtraction angiogram (lateral view) shows a dural carotid-cavernous fistula drained anteriorly and posteriorly with a combination of extra cranial internal carotid aneurysm.

A 21-year-old man was admitted to the hospital with left eye redness and swelling, mild blurred, and double vision. He experienced nausea and visual deterioration after head trauma a month earlier. He denied a history of diabetes and hypertension. There was no history of any infectious diseases and no history of fever, sickness, or any surgery. He was a non-smoker without allergies to any medications. An ophthalmic examination showed left eye proptosis, severe chemosis, lateral rectus palsy, and high intraocular pressure (thereafter – IOP; Figures 1A,B). Visual acuity (thereafter – VA) was 10/10 in the right eye and 1/10 in the left eye.

### 2.2. Case 2

A 27-year-old man was admitted to the hospital with head trauma resulting from a 3-meter fall. After 5 days, he developed redness in his right eye with diplopia. He had no history of diabetes or hypertension, but he was a smoker. An ophthalmic examination showed proptosis, chemosis, and lateral rectus palsy in the right eye. His visual acuity was 10/10 in the left eye and 3/10 in the right eye.

### 2.3. Diagnostic procedures

**Figure 2:**
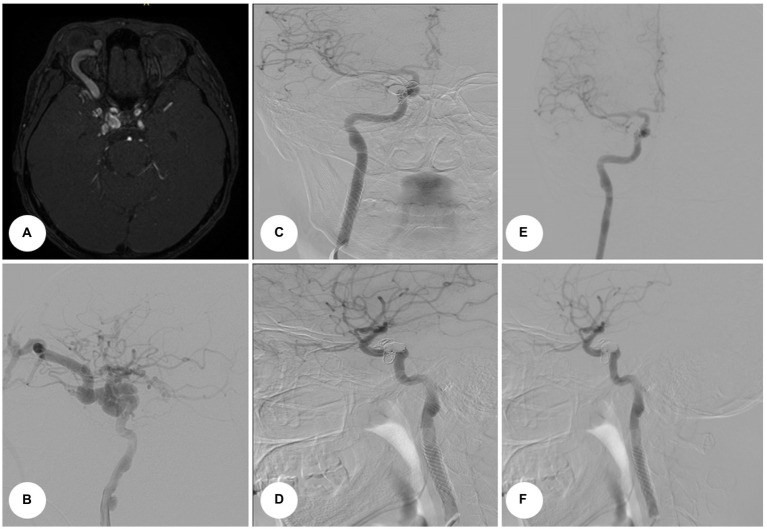
An instrumental examination of Case 2. **(A)** Axial MRI revealed enlargement of the superior ophthalmic vein and expansion of the right cavernous sinus in a patient with right side carotid-cavernous fistula. **(B)** Preoperative angiogram. **(C)** Postoperative control angiograms reveal stent deployment and aneurysm exclusion from the blood circulation. Anterior–posterior projection. **(D)** Lateral view. **(E)** Post-procedural angiograms (follow-up after 6 months). Anterior view. **(F)** Post-procedural angiograms (follow-up after 6 months). Lateral view.

In both cases, magnetic resonance imaging (thereafter – MRI) revealed enlarged cavernous sinuses and enlarged superior and inferior ophthalmic veins (Figures 1C, 2A). Angiograms confirmed the direct left (first case) and right (second case) CCF between the internal carotid artery (thereafter – ICA) and the cavernous sinus by demonstrating rapid filling of the cavernous sinus. Cavernous sinus drained anteriorly through both ophthalmic veins, and posteriorly through the inferior petrosal sinus. Moreover, dissecting aneurysms at the extracranial part of the left (first case) and right (second case) ICAs were detected (Figures 1D, 2B).

### 2.4. Treatment

**Figure 3:**
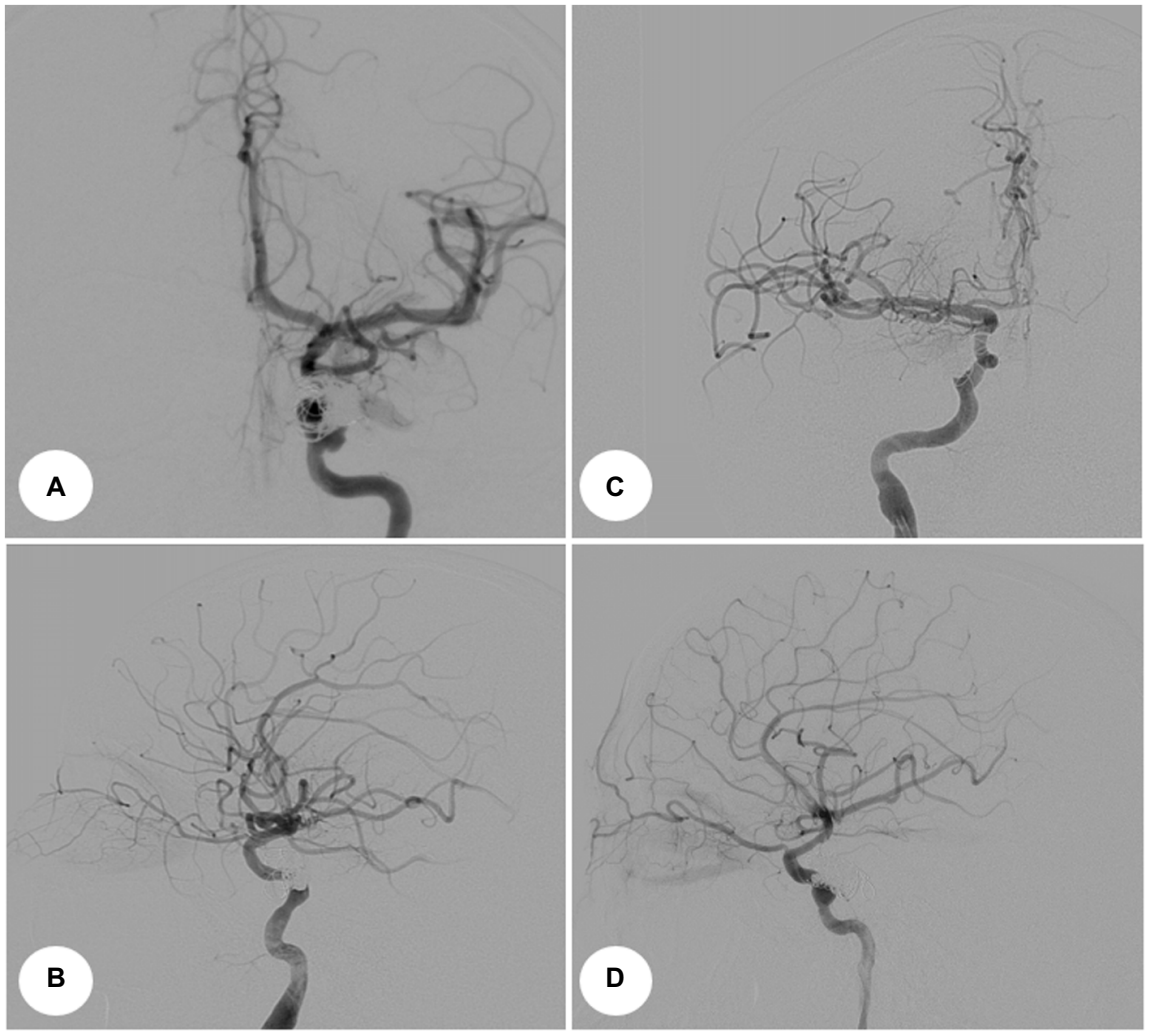
Intraoperative angiograms. Digital subtraction angiograms from internal carotid artery. **(A)** Anterior–posterior projection view, Case 1. **(B)** Lateral view, Case 1. **(C)** Anterior–posterior projection view, Case 2. **(D)** Lateral view, Case 2.

**Figure 4:**
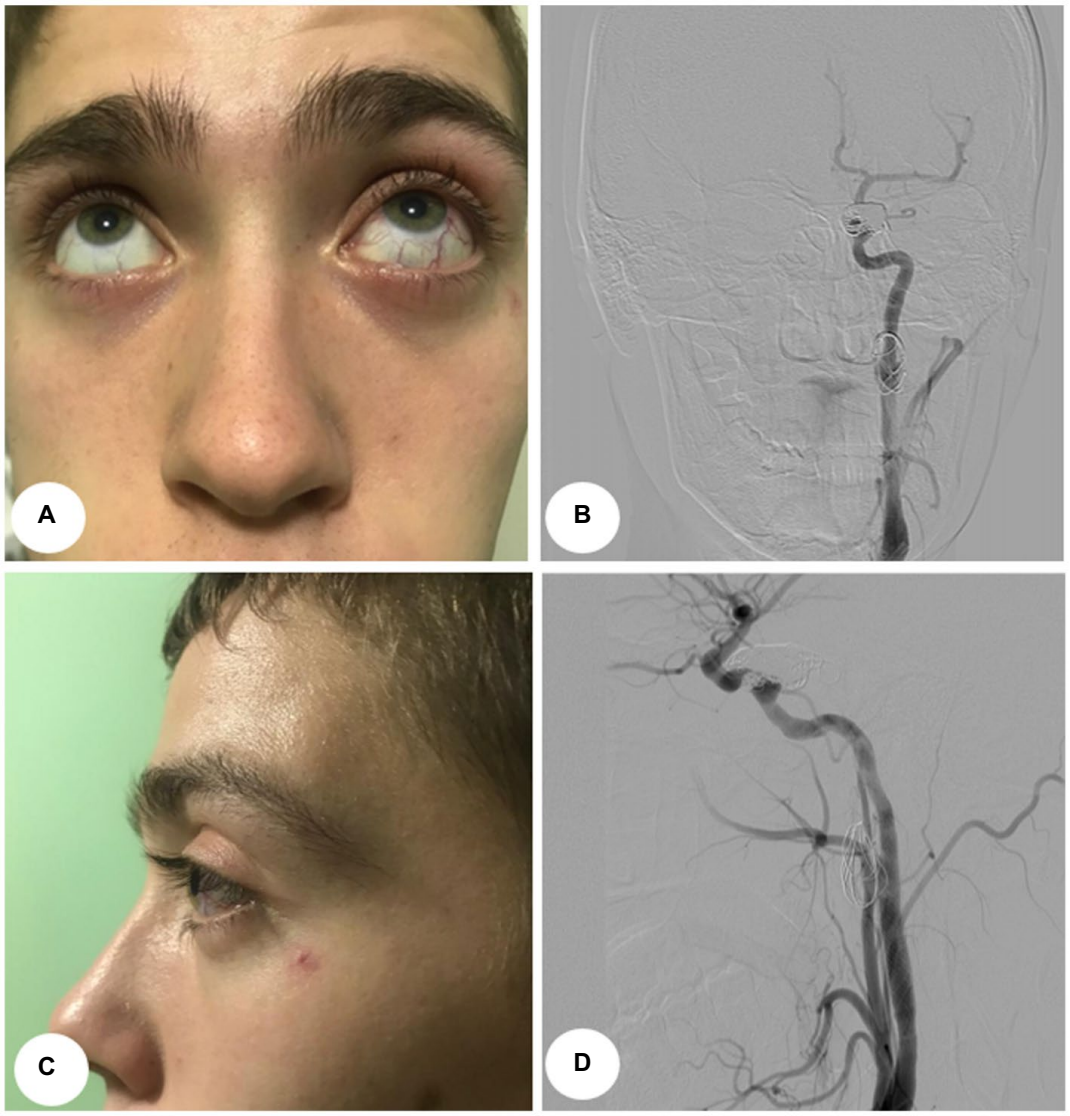
Postoperative images, Case 1. **(A)** Anterior view. **(B)** Lateral view. **(C)** Postoperative control angiograms show complete occlusion of the CCFs with coil mass. Anterior–posterior projection view. **(D)** Lateral view.

Procedures for both cases were identical. First, embolization of CCF with balloon-assisted coiling was performed under general endotracheal anesthesia. Right femoral access was obtained using a 7-Fr sheath on the arterial side. A 7-Fr guide catheter was placed at the ICA, and the AV fistula was catheterized using an Echelon 10 (ev3, Inc.) as a conduit to the cavernous sinus. An angiogram was performed to ascertain that the microcatheter was positioned proximally to the origin of the SOV. The patient was administered heparin intravenously (5,000 U). At this point, a Hyperglide balloon (ev3, Inc.) was navigated on the arterial side and was left in the position spanning the fistulous site. The balloon was subsequently inflated and coils were deployed in the cavernous sinus point to prevent coil herniation into the ICA. The balloon was deflated and the angiogram showed no early venous drainage from the arterial injection. The microcatheter was removed, and a control angiogram was obtained. It was compared with the initial angiogram to rule out embolic events and confirm complete fistula obliteration (Figure 3). Proptosis and chemosis resolved the next day in both patients (Figures 4A,B).

The second procedure was performed 3 months later. The patient was under dual antiplatelet therapy [clopidogrel 300 mg, acetylsalicylic acid (thereafter – ASA) 500 mg]. The endovascular procedure was performed under local anesthesia with continuous hemodynamic monitoring. The ICA access was obtained through the right femoral artery. After the femoral artery was accessed 5,000 U of heparin was administered. A guide catheter using a 0.035-inch stiff guidewire was placed in the common carotid artery (thereafter – CCA). After the placement of the soft guidewire past the lesion, a self-expanding bare stent Casper 7 × 30 mm (Terumo Co., Tokyo, Japan) was placed. The stent was expanded 1cm proximal and distal to the aneurysm and was used for the landing zones of the stent-grafts. In the case of the first patient, the aneurysm was embolized by stent-assisted coiling (2 coils). Multiple control angiograms showed the exclusion of the aneurysm from the blood circulation (Figures 2C,D, 4C,D). Clopidogrel was discontinued after 6 months, while ASA was administered continuously. Control angiography was performed 6 months later on one patient (Figures 2E,F) and revealed complete occlusion of ECAA and CCF. The second patient was unable to undergo control angiography due to financial restraints. However, the patient reported no complaints during the telephone interview.

## 3. Discussion

Although some reports about ICA aneurysms with CCF exist (5), and literature on CCF and ECAA separately (6, 7), to our knowledge, this is the first study to present cases of CCF and ECAA combination occurring in a patient. Trauma is the most common cause of direct CCF and dissecting aneurysm. In two cases presented here, patients reported a history of trauma, confirming the recommendation to ask cases of single-hole dural fistula about trauma in case of suspicion of direct CCF (8). A treatment protocol for the two pathologies combination is not established.

In the presented cases, CCF was treated first. It reduced the risk of CCF resulting in hemorrhage from dual antiplatelet therapy. The resulting blood thinning could make the occlusion of CCF slower and more difficult with more materials used due to slow thrombosis. CCF treatment would relieve patients’ acute symptoms.

Different options for CCFs treatment are available, such as conservative management and embolization. Conservative management can be effective in approximately 30% of indirect CCF and 17% of direct CCF cases, but most of these cases had mild clinical presentations (9).

Detachable balloon occlusion is a cost-effective and straightforward option for treating CCFs, but it can lead to complications such as cerebral infarction or CCF recurrence (10). Embolization with the ethylene-vinyl alcohol copolymer Onyx is another treatment option for CCFs. However, using a combination of dimethyl sulfoxide and Onyx can be toxic and pose a potential risk for trigeminocardiac reflex-induced bradycardia (11).

Placement-covered or flow-diverting stents are also used as an alternative method for the treatment of CCFs. Despite dramatic initial improvement, delayed thrombosis remains a problem with the use of currently available covered stents in the cerebral circulation. In the cases presented, balloon-assisted embolization was chosen as the treatment method due to its advantages, such as the lack of complications, practicality, and ability to keep the ICA unobstructed.

The second surgical intervention was ECAA treatment. Surgical approaches include microsurgical resection of the aneurysm, with arterial reconstruction, and endovascular stenting (12, 13). In presented cases, the basis for choosing the latter was the prevention of cranial nerve injuries, which can occur during open surgical repair. Aneurysms near the neck often form from dissection, therefore; stent placement is optimal for the increase of vessel endothelialization. From the self-expandable carotid stent and flow diverter, the former was chosen due to its higher stent cell density, and cost-effectiveness, and demonstrated equal outcomes to the flow diverter (14).

## 4. Limitation

The main limitation of this study is the lack of enough cases. We cannot provide controlled clinical research to demonstrate the availability and efficacy of our strategy. However, follow-up results show a good outcome after 6 months.

## 5. Conclusion

The presented cases showed that aneurysm-associated CCF is a complex and risky pathology. Evaluation of the fistula and aneurysm angioarchitecture and digital subtraction angiography is essential. This case report suggests a treatment plan, and multi-step endovascular surgery when encountering CCF and ECAA combination in a patient.

## Data availability statement

The original contributions presented in the study are included in the article/Supplementary material, further inquiries can be directed to the corresponding author.

## Ethics statement

The studies involving human participants were reviewed and approved by The Institutional Review Board of the National Centre for Neurosurgery. The patients/participants provided their written informed consent to participate in this study. Written informed consent was obtained from the individual(s) for the publication of any potentially identifiable images or data included in this article.

## Author contributions

All authors contributed to the study’s conception and design. Material preparation and data collection were performed by CN and YM. CN and AK wrote the first draft of the manuscript. SA wrote the final version of the manuscript. All authors commented on previous versions of the manuscript. All authors contributed to the article and approved the submitted version.

## Conflict of interest

The authors declare that the research was conducted in the absence of any commercial or financial relationships that could be construed as a potential conflict of interest.

## Publisher’s note

All claims expressed in this article are solely those of the authors and do not necessarily represent those of their affiliated organizations, or those of the publisher, the editors and the reviewers. Any product that may be evaluated in this article, or claim that may be made by its manufacturer, is not guaranteed or endorsed by the publisher.
